# Mid-term clinical and echocardiographic results of the INSPIRIS RESILIA aortic valve: a retrospective comparison to the Magna Ease

**DOI:** 10.1093/icvts/ivad117

**Published:** 2023-07-18

**Authors:** Jérémy Bernard, Gabriel Georges, Sébastien Hecht, Philippe Pibarot, Marie-Annick Clavel, Shervin Babaki, Dimitri Kalavrouziotis, Siamak Mohammadi

**Affiliations:** Cardiology Division, Institut universitaire de cardiologie et de pneumologie de Québec — Université Laval/Quebec Heart and Lung Institute — Laval University, Quebec, QC, Canada; Cardiac Surgery Division, Institut universitaire de cardiologie et de pneumologie de Québec — Université Laval/Quebec Heart and Lung Institute — Laval University, Quebec, QC, Canada; Cardiology Division, Institut universitaire de cardiologie et de pneumologie de Québec — Université Laval/Quebec Heart and Lung Institute — Laval University, Quebec, QC, Canada; Cardiology Division, Institut universitaire de cardiologie et de pneumologie de Québec — Université Laval/Quebec Heart and Lung Institute — Laval University, Quebec, QC, Canada; Cardiology Division, Institut universitaire de cardiologie et de pneumologie de Québec — Université Laval/Quebec Heart and Lung Institute — Laval University, Quebec, QC, Canada; Research Division, Institut universitaire de cardiologie et de pneumologie de Québec — Université Laval/Quebec Heart and Lung Institute — Laval University, Quebec, QC, Canada; Cardiac Surgery Division, Institut universitaire de cardiologie et de pneumologie de Québec — Université Laval/Quebec Heart and Lung Institute — Laval University, Quebec, QC, Canada; Cardiac Surgery Division, Institut universitaire de cardiologie et de pneumologie de Québec — Université Laval/Quebec Heart and Lung Institute — Laval University, Quebec, QC, Canada

**Keywords:** Surgical aortic valve replacement, INSPIRIS RESILIA, Magna Ease, Patient–prosthesis mismatch, Structural valve deterioration, Transvalvular gradient

## Abstract

**OBJECTIVES:**

The INSPIRIS aortic valve combines the RESILIA proprietary tissue preservation process and an expandable stent frame to benefit future transcatheter valve-in-valve procedures. As the INSPIRIS valve became commercially available in 2017, mid-term outcome reports are scarce. We aimed to evaluate mid-term safety and echocardiographic performance of the INSPIRIS valve in comparison to its predecessor, the Carpentier Edwards Perimount Magna Ease (ME).

**METHODS:**

This study was a retrospective single-centre study. Clinical results included early postoperative outcomes, mid-term mortality and readmission for cardiovascular cause or stroke. Echocardiographic follow-up (FU) was performed at discharge and 1–3, 6, 12 and 24 months. Clinical end point analyses were accomplished with a propensity score matching analysis and FU echocardiographic data comparisons using pairwise analyses and linear mixed-effect models.

**RESULTS:**

We included 953 patients who received an INSPIRIS (*n* = 488) or ME (*n* = 463) bioprosthesis between January 2018 and July 2021. In the matched population (*n* = 217 per group), no significant difference in short-term outcomes was observed, survival was similar at 30 months (INSPIRIS: 94% vs ME: 91%, *P* = 0.89), but freedom from readmission was higher in the INSPIRIS group (94% vs 86%, *P* = 0.014). INSPIRIS valves had a lower gradient at discharge (∼10 vs 14 mmHg, *P* < 0.001), 1–3 months (∼10 vs 12 mmHg, *P* < 0.001) and 24 months (∼11 vs 17 mmHg, *P* < 0.001) in paired analyses and significantly lower evolution of mean transvalvular gradients compared to ME.

**CONCLUSIONS:**

This study represents the largest comparative evaluation of the INSPIRIS to the ME valves, which demonstrated safe clinical outcomes and favourable haemodynamic performance at 2 years. Long-term FU is underway.

**Figure:**
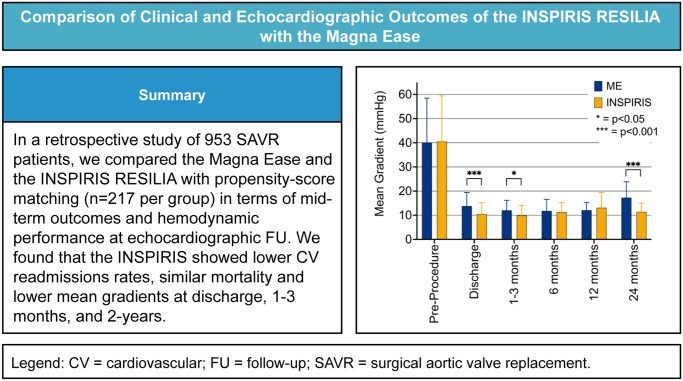

## INTRODUCTION

In patients undergoing surgical aortic valve replacement (SAVR), choosing a bioprosthetic tissue valve avoids the need for life-long anticoagulation at the cost of potential structural valve deterioration (SVD) and reintervention. In some cases, valve-in-valve (VinV) transcatheter aortic valve replacement (TAVR) may provide a lower-risk alternative to redo-SAVR, further tipping the preference towards the choice for a bioprosthetic valve [1]. However, not all patients will become candidates for VinV therapy and the ideal strategy for lifetime management of aortic stenosis remains unknown. In young patients, risks of permanent pacemaker implantation, paravalvular leaks, surgical risk of TAVR explantation and limited percutaneous experience in patients with bicuspid aortic valve may favour a SAVR-first strategy [1–4]. To reduce the risks of reintervention and improve patient outcomes, there is a need for surgical bioprosthetic valves with improved durability and performance.

The INSPIRIS RESILIA Aortic Valve (INSPIRIS; Edwards Lifesciences Corporation, Irvine, CA, USA) is a stented tri-leaflet bovine pericardial tissue valve for SAVR. The valve was made commercially available in 2017 and integrates 2 technological advances: (i) an expandable stent frame to minimize gradients of future VinV and reduce potential prosthesis–patient mismatch (PPM) and (ii) the RESILIA Tissue Integrity Preservation anti-calcification process to reduce leaflet calcification and risk of SVD.

While initial clinical investigation of the RESILIA tissue has provided encouraging results, reports of the INSPIRIS valve are limited to observational studies [5–7]. Considering previous early failures of new bioprosthetic valves and the conceptual advantages of the INSPIRIS valve which favours its utilisation in younger patients, close clinical and haemodynamic performance follow-up (FU) is paramount [8]. We aimed to compare mid-term clinical and echocardiographic outcomes of patients who received an INSPIRIS valve to those of patients who were implanted with a Carpentier Edwards Perimount Magna Ease (ME; Edwards Lifesciences Corporation, Irvine, CA, USA) valve during the same period.

## PATIENTS AND METHODS

### Ethical statement

The study was approved by the local Ethics Committee (Quebec Heart and Lung Institute, 31 August 2022, # 20831) and, due to its retrospective nature, patient consent was waived.

### Study population

We retrospectively interrogated our institution’s electronic medical records for patients who underwent SAVR with the INSPIRIS valve from the time of the first INSPIRIS implant in our centre in January 2018 until the end of July 2021. A comparison group was formed from patients who had received a ME valve in our institution during the same timeframe. Included were patients 18 years or older who underwent elective primary SAVR, isolated or in combination with coronary artery bypass graft, aortic root surgery or other valvular repair or replacement. Patients with active or recent (<3 months) endocarditis, redo-procedures, patients with prior transcatheter aortic valve replacement and urgent procedures were excluded.

### Procedure and follow-up

The decision on bioprosthesis valve type was individualized and based on patient preference, and left completely at the discretion of the surgeon. SAVR was performed via median sternotomy in all patients except for 2 who underwent minimally invasive procedures (i.e. not included in matched analyses). All patients were maintained at least on a single antiplatelet therapy with a daily low-dose of aspirin initiated in the early postoperative period, depending on concomitant procedures and comorbidities. Patients did not receive a vitamin-K antagonist following SAVR unless they underwent concomitant mitral valve surgery or had other indication for anticoagulation with a vitamin-K antagonist. The choice of antithrombotic regimen was subject to change at the discretion of the surgeon or referring cardiologist during FU. Routine FU included clinical evaluation and transthoracic echocardiography at discharge, 1–3, 6 and 12 months and then yearly. Patients completed their FU in our centre or referring hospital based on geographic distribution and efforts were deployed to retrieve external echocardiographic results.

### Clinical and echocardiographic parameters

Baseline patient characteristics including clinical, laboratory and echocardiography data were obtained, and operative risk was assessed using the European System for Cardiac Operative Risk Evaluation II criteria. Intraoperative data included valve type and size, concomitant procedures, bypass and cross-clamp time. Operative mortality and postoperative outcomes were documented up to 30 days. All-cause mortality, readmissions for cardiovascular causes or stroke/transient ischaemic attack and aortic valve reinterventions were collected for the duration of FU. Echocardiographic outcomes were defined per the Valve Academic Research Consortium-3 criteria [9]. Degree of measured PPM was evaluated at 1–3 months with the echocardiographic measured effective orifice area (EOA) and categorized as mild/no PPM for EOA indexed to body surface area (EOAi) >0.85 cm^2^/m^2^, moderate for EOAi >0.65 cm^2^/m^2^ but ≤0.85 cm^2^/m^2^ and severe for EOAi ≤0.65 cm^2^/m^2^. In patients with a body mass index >30 kg/m^2^, the cutoffs were: mild >0.70 cm^2^/m^2^, moderate >0.55 but ≤0.70 cm^2^/m^2^ and severe ≤0.55 cm^2^/m^2^.

### Study end points

Clinical end points included 30-day mortality and postoperative adverse events, aortic valve reintervention and mid-term incidence of readmission for cardiovascular causes or stroke/transient ischaemic attack and death. Short-term outcomes were assessed with our internal database and mid-term by an in-depth interrogation of the provincial institution (Institut de la statistique du Québec) central database. Echocardiographic end points were mean transvalvular gradients (MG), EOAi, incidence of SVD and degree of PPM.

### Statistical analysis

This manuscript follows the *European Journal of Cardio-Thoracic Surgery* and the *Interactive CardioVascular and Thoracic Surgery* guidelines for statistical and data reporting [10]. Results are expressed as standard descriptive statistics: absolute and relative frequencies for categorical variables and mean (standard deviation) or median and interquartile range (IQR) for continuous data. Unmatched data were compared between bioprosthesis groups using chi-squared or the Fisher exact test for categorical variables and the Student's *t*-test or Mann–Whitney test for continuous variables, as appropriate. Matched data were compared with McNemar, paired *t*-test or Wilcoxon signed rank test, as appropriate. Standardized mean differences were calculated to examine the characteristic distributions between groups after 1:1 propensity score matching (PSM). Standardized mean differences >0.15 or <–0.15 was considered imbalance. Early and mid-term clinical end points were assessed in the PSM cohort. Mid-term outcomes were depicted in Kaplan–Meier curves and compared with the stratified log-rank analyses. Univariate/multivariate Cox analyses were used to test the association of the ME prosthesis with the study end points after PSM. Evolution of MG and EOAi were assessed in the PSM cohort with pairwise analysis to weight for incomplete data. Linear mixed-effect models were also used to show and compare the evolution of both main haemodynamic performance variables (i.e. EOAi, MG) during FU [11]. The PSM has already been described elsewhere [12]. Ours included valve size, age, sex, body surface area, body mass index and left ventricle ejection fraction at baseline. Incidence of SVD and PPM was assessed in the PSM cohort with available echocardiographic FU. Changes between FUs were assessed using paired analysis. Data were analysed using SPSS software (V.28, IBM, Chicago, IL, USA) and Prism (GraphPad Software, San Diego, CA, USA). All measures were tested at a significance level of 0.05.

## RESULTS

### Patient characteristics

The study design is presented in Supplementary Material, Fig. S1. Of the 1460 adult patients who underwent elective SAVR between January 2018 and July 2021, 488 patients received and INSPIRIS valve and 465 received a ME bioprosthesis. Of those 953 patients, 745 (78%) patients had at least 1 postoperative echocardiographic FU. This included 393 patients in the INSPIRIS group (median FU: 15.0 [IQR: 5.9–23.4] months) and 352 in the ME group (median FU: 17.5 [IQR: 7.0–25.3]). Baseline clinical and echocardiographic characteristics of the unmatched and matched population (*n* = 217 in each group) are presented in Table 1. In the unmatched population, patients in the INSPIRIS group were younger, with a mean age of 67 (7) vs 74 (7) years old in the ME group, and 83 (17%) vs 24 (5%) aged under 60, respectively (*P* < 0.001). Approximately 73% of all patients were male. Coronary artery disease and atrial fibrillation were more common in the ME group (coronary artery disease: 55 vs 48%, *P* = 0.03, atrial fibrillation: 24 vs 14%, *P* < 0.001). Patients in the INSPIRIS group were at lower operative risk (European System for Cardiac Operative Risk Evaluation II: 1.8 [1.2–3.3] vs 3.2 [1.8–6.2]%, *P* < 0.001). Left ventricle ejection fraction was preserved in both groups (INSPIRIS: 57 (10)%, ME: 55 (11)%, *P* = 0.35). Left ventricular outflow tract diameter was similar between groups (INSPIRIS: 22.3 (2.2) mm, ME: 22.7 (2.7) mm, *P* = 0.08). All patients had severe aortic stenosis requiring intervention. All baseline characteristics were similar between groups in the matched population aside for unbalanced systolic pulmonary artery pressure, LVOT diameter, ascending aorta diameter and LVSV (Table 1), but it can be explained by important missing values in these variables.

**Table 1:** Baseline clinical and echocardiographic characteristics

Variables	Unmatched cohort				Matched cohort			
	All patients (*N* = 953)	ME (*n* = 465; 49%)	INSPIRIS (*n* = 488; 51%)	*P-*Value	ME (*n* = 217)	INSPIRIS (*n* = 217)	*P*-Value	SMD
Clinical								
Age (years)	70 (8)	74 (7)	67 (7)	**<0.001**	70 (7)	69 (7)	0.26	0.143
Men, *n* (%)	693 (73)	348 (75)	345 (71)	0.15	154 (71)	159 (73)	0.67	0.045
Body surface area (m^2^)	1.90 (0.22)	1.88 (0.22)	1.91 (0.22)	0.06	1.89 (0.24)	1.90 (0.20)	0.66	−0.045
Body mass index (kg/m^2^)	28.6 (5.3)	28.1 (5.1)	29.0 (5.5)	**0.004**	28.5 (5.6)	28.5 (5.0)	0.94	0.000
Hypertension, *n* (%)	753 (79)	381 (82)	372 (76)	**0.03**	166 (76.5)	172 (79.3)	0.53	−0.068
Obesity, *n* (%)	332 (35)	142 (31)	190 (39)	**0.007**	73 (33.6)	82 (37.8)	0.41	−0.088
Dyslipidaemia, *n* (%)	787 (83)	383 (82)	404 (83)	0.86	177 (81.6)	185 (85.3)	0.40	−0.099
Diabetes, *n* (%)	294 (31)	138 (30)	156 (32)	0.43	64 (29.5)	67 (31)	0.84	−0.033
Chronic kidney disease, *n* (%)	22 (2)	13 (3)	9 (2)	0.33	5 (2.3)	6 (2.8)	1.00	−0.032
Smoking,^a^ *n* (%)	500 (53)	234 (50)	266 (55)	0.20	119 (54.8)	102 (47)	0.12	0.157
COPD, *n* (%)	75 (8)	43 (9)	32 (7)	0.12	20 (9.2)	11 (5.1)	0.14	0.160
Coronary artery disease, *n* (%)	488 (51)	255 (55)	233 (48)	**0.03**	102 (47)	107 (49.3)	0.71	−0.046
History of MI, *n* (%)	183 (19)	104 (22)	79 (16)	**0.02**	41 (18.9)	32 (14.7)	0.31	0.113
History of PCI, *n* (%)	109 (11)	59 (13)	50 (10)	0.24	21 (9.7)	22 (10.1)	1.00	−0.013
Atrial fibrillation, *n* (%)	181 (19)	111 (24)	70 (14)	**<0.001**	36 (16.9)	37 (17.1)	1.00	−0.005
Cerebrovascular disease, *n* (%)	88 (9)	48 (10)	40 (8)	0.26	22 (10.1)	18 (8.3)	0.61	0.062
Peripheral vascular disease, *n* (%)	90 (9)	40 (9)	50 (10)	0.39	18 (8.3)	25 (11.5)	0.32	−0.107
Pulmonary hypertension, *n* (%)	36 (4)	26 (6)	10 (2)	**0.004**	10 (4.9)	6 (2.8)	0.45	−0.109
Permanent pacemaker, *n* (%)	48 (5)	36 (8)	12 (3)	**<0.001**	14 (6.5)	8 (3.7)	0.26	0.128
NYHA class III–IV, n (%)	298 (31)	154 (33)	144 (30)	0.23	151 (69.6)	156 (71.9)	0.60	−0.051
EuroSCORE II (%)	2.5 (1.4–4.6)	3.2 (1.8–6.2)	1.8 (1.2–3.3)	**<0.001**	2.5 (1.4–4.4)	1.9 (1.3–3.5)	0.44	0.071
Medication								
ACE/ARA, *n* (%)	539 (57)	269 (58)	270 (55)	0.43	125 (57.6)	122 (56.2)	0.84	0.028
Antiplatelets, *n* (%)	615 (65)	307 (66)	308 (63)	0.35	139 (64.1)	140 (64.5)	1.00	−0.008
Aspirin, *n* (%)	596 (63)	297 (64)	299 (61)	0.41	133 (61.3)	134 (61.8)	1.00	−0.010
Beta-blockers, *n* (%)	459 (48)	230 (50)	229 (47)	0.43	100 (46.1)	101 (46.5)	1.00	−0.008
Calcium blockers, *n* (%)	331 (35)	168 (36)	163 (33)	0.38	76 (35)	79 (36.4)	0.85	−0.029
Anti-lipids, *n* (%)	725 (76)	361 (78)	364 (75)	0.27	167 (77)	170 (78.3)	0.82	−0.031
Diuretics, *n* (%)	339 (36)	182 (39)	157 (32)	**0.03**	79 (36.4)	69 (31.8)	0.35	0.097
Anticoagulant, *n* (%)	218 (23)	123 (27)	95 (20)	**0.01**	49 (22.6)	41 (18.9)	0.38	0.091
Antiarrhythmics, *n* (%)	56 (6)	36 (8)	20 (4)	**0.02**	16 (7.4)	8 (3.7)	0.15	0.162
Laboratory data								
Creatinine (µmol/l)	81 (69–94)	83 (71–98)	79 (68–91)	**<0.001**	79 (68–93)	81 (68–92)	0.87	0.019
Renal clearance (Cock) (ml/min)	71 (57–89)	65 (53–82)	78 (63–94)	**<0.001**	74 (58–91)	75 (59–90)	0.82	0.000
Doppler echocardiography data								
IVSd (mm)	11.1 (3.7)	10.9 (2.9)	10.9 (2.8)	0.99	11.1 (2.8)	11.0 (2.8)	0.61	0.035
PWd (mm)	10.8 (3.3)	10.9 (2.3)	10.6 (2.2)	**0.03**	11.0 (2.3)	10.9 (2.2)	0.91	0.044
LVEDD (mm)	47.9 (7.2)	48.2 (7.6)	47.7 (6.8)	0.41	48.0 (7.6)	48.1 (7.3)	0.93	−0.013
LVESD (mm)	31.1 (8.6)	31.4 (8.4)	30.9 (8.7)	0.22	30.9 (8.2)	31.3 (9.4)	0.70	−0.045
LV mass index (g/m^2^)	106 (53)	107 (34)	106 (66)	**0.04**	108 (35)	108 (77)	0.97	0.000
LVOT diameter (mm)	22.5 (2.4)	22.7 (2.7)	22.3 (2.2)	0.08	22.5 (3.2)	21.7 (2.0)	**0.01**	0.300
Ascending aorta diameter (mm) (*n* = 735)	37.4 (6.5)	37.9 (6.7)	36.9 (5.9)	**0.008**	37.2 (6.3)	35.2 (3.9)	0.12	0.382
LVSV (ml) (*n* = 821)	76 (21)	75 (22)	76 (20)	0.52	78 (25)	66 (16)	**<0.001**	0.572
LVSV index (ml/m^2^) (*n* = 821)	40 (11)	41 (13)	40 (10)	0.58	42 (12)	35 (9)	**<0.001**	0.660
LVEF (%)	56.0 (10.5)	55.0 (11.1)	57.0 (9.7)	**<0.001**	57.2 (9.8)	56.2 (10.6)	0.35	0.098
SPAP (mmHg) (*n* = 700)	41 (15)	43 (16)	38 (12)	**0.002**	43 (17)	38 (12)	**0.02**	0.340
Etiology of aortic valve disease				**<0.001**			0.23	–
Calcification, *n* (%)	629 (66)	337 (73)	292 (60)		147 (67.7)	138 (63.6)		0.086
Congenital, *n* (%)	282 (30)	101 (22)	181 (37)		57 (26.3)	74 (34.1)		−0.171
Rheumatism, *n* (%)	22 (2)	12 (3)	10 (2)		7 (3.2)	2 (0.9)		0.163
Other, *n* (%)	20 (2)	15 (3)	5 (1)		6 (2.8)	5 (1.4)		0.098
Severity of aortic stenosis								
Peak aortic jet velocity (m/s)	3.94 (0.98)	3.84 (1.01)	4.04 (0.94)	**0.004**	3.94 (0.99)	3.96 (0.96)	0.79	−0.021
Mean gradient (mmHg)	40 (19)	39 (18)	42 (19)	**0.02**	40 (19)	40 (19)	0.81	0.000
Indexed AVA (cm^2^/m^2^)	0.49 (0.23)	0.50 (0.26)	0.47 (0.21)	0.05	0.50 (0.25)	0.48 (0.22)	0.37	0.085
Concomitant VHD								
≥ Moderate AR, *n* (%)	253 (27)	136 (30)	117 (24)	0.07	62 (28.6)	57 (26.3)	0.67	0.052
≥ Moderate MR, *n* (%)	127 (13)	83 (18)	44 (9)	**<0.001**	34 (15.7)	25 (11.5)	0.25	0.123
≥ Moderate TR, *n* (%)	65 (7)	46 (10)	19 (4)	**<0.001**	17 (7.8)	14 (6.5)	0.69	0.050

Values are represented as mean (SD), median (interquartile range) or *n* (%). Text in bold highlights the statistically significant differences between the 2 groups.

a Smoking: current or history of smoking.

ACE: angiotensin-conversing enzyme inhibitors; AR: aortic regurgitation; ARA: angiotensin receptor antagonists; AVA: aortic valve area; COPD: chronic obstructive pulmonary disease; EuroSCORE II: European System for Cardiac Operative Risk Evaluation II; INSPIRIS: INSPIRIS RESILIA; IVSd: interventricular septum diameter; LV: left ventricular; LVEDD: LV end-diastolic diameter; LVEF: LV ejection fraction; LVESD: LV end-systolic diameter; LVOT: LV outflow track; LVSV: LV stroke volume; ME: Carpentier Edwards Magna Ease; MI: myocardial infarction; MR: mitral regurgitation; NYHA: New York Heart Association; PCI: percutaneous coronary intervention; PWd: posterior wall diameter; SMD: standardized mean differences; SPAP: systolic pulmonary artery pressure; TR: tricuspid regurgitation; VHD: valvular heart disease.

### Procedure data and early postoperative outcomes

Operative data of both unmatched and matched populations are presented in Table 2. Patients in the ME group were more likely to undergo a combined procedure (75 vs 63%, *P* < 0.001) of which coronary artery bypass graft was the most frequent (49% overall). There were 13 and 11% of concomitant ascending aorta replacement in the ME and INSPIRIS groups, respectively (*P* = 0.33). Concomitant mitral valve replacement was more common in patients receiving an ME (12 vs 6%, *P* = 0.002). As predicted, bypass and cross-clamp times were slightly longer in the ME group (bypass time: 103 [77–131] vs 91 [72–122] min, *P* < 0.001 and cross-clamp time: 82 [59–107] vs 71 [56–97] min, *P* = 0.002, respectively). Implanted valve sizes were similar for both bioprostheses (*P* = 0.07). Size 23 mm was the most frequent (37% overall), followed by 25 mm (28% overall) and 21 mm (20% overall). Only 2% of patients in both groups received a 19-mm valve. Postoperative outcomes in matched population are presented in Table 3. There were no significant differences in early postoperative outcomes between groups in the matched population (Table 3). Early adverse events stratified by isolated AVR are presented in Supplementary Material, Table S1.

**Table 2:** Operative data

Variables	Unmatched cohort				Matched cohort			
	All patients (*N* = 953)	ME (*n* = 465; 49%)	INSPIRIS (*n* = 488; 51%)	*P-*Value	ME (*n* = 217)	INSPIRIS (*n* = 217)	*P-*Value	SMD
Isolated AVR, *n* (%)	299 (31)	117 (25)	182 (37)	**<0.001**	61 (28)	84 (39)	**0.03**	−0.235
Bentall procedure, *n* (%)	16 (2)	11 (2)	5 (1)	0.11	8 (4)	3 (1)	0.23	0.193
Concomitant CABG, *n* (%)	462 (49)	243 (52)	219 (45)	**0.02**	100 (46)	101 (47)	1.00	−0.020
Other concomitant surgery								
Ascending aorta replacement	113 (12)	60 (13)	53 (11)	0.33	32 (15)	21 (12)	0.15	0.088
Aortic arch replacement	33 (4)	15 (3)	18 (4)	0.70	10 (5)	6 (3)	0.45	0.102
Aortoplasty	46 (5)	21 (5)	25 (5)	0.66	7 (3)	7 (3)	1.00	0.000
Root sinus enlargement	57 (6)	31 (7)	26 (5)	0.38	15 (7)	9 (4)	0.29	0.132
MVR, *n* (%)	88 (9)	57 (12)	31 (6)	**0.002**	26 (12)	14 (7)	0.06	0.171
Tricuspid valve intervention, *n* (%)	21 (2)	13 (3)	8 (2)	0.22	6 (3)	8 (4)	0.77	−0.054
Maze procedure, *n* (%)	42 (4)	24 (5)	18 (4)	0.27	11 (5)	8 (4)	0.65	0.048
Cardiopulmonary bypass time (min)	97 (74–126)	103 (77–131)	91 (72–122)	**<0.001**	98 (77–129)	91 (71–125)	0.08	0.178
Aortic cross-clamp time (min)	77 (57–102)	82 (59–107)	71 (56–97)	**0.002**	78 (59–107)	70 (54–98)	0.09	0.192
Prosthesis size				0.07			0.22	
19 mm	19 (2)	10 (2)	9 (2)		9 (4)	2 (1)		0.193
21 mm	190 (20)	79 (17)	111 (23)		42 (19)	47 (22)		−0.074
23 mm	354 (37)	167 (36)	187 (38)		86 (40)	74 (34)		0.125
25 mm	264 (28)	139 (30)	125 (26)		52 (24)	61 (28)		−0.091
27 mm	106 (11)	56 (12)	50 (10)		20 (10)	30 (14)		−0.123
29 mm	20 (2)	14 (3)	6 (1)		8 (4)	3 (1)		0.193

Values are represented as median (interquartile range) or *n* (%). Text in bold highlights the statistically significant differences between the 2 groups.

AVR: aortic valve replacement; CABG: coronary artery bypass grafting; INSPIRIS: INSPIRIS RESILIA; ME: Carpentier Edwards Magna Ease; MVR: mitral valve replacement; SMD: standardized mean differences.

**Table 3:** Postoperative outcomes at 30 days

Variables	Matched cohort			
	ME (*n* = 217)	INSPIRIS (*n* = 217)	*P-*Value	SMD
Adverse events				
In-hospital mortality, *n* (%)	5 (2.3)	6 (2.8)	1.00	−0.032
Stroke/TIA, *n* (%)	5 (2.3)	4 (1.8)	1.00	0.035
Cardiogenic shock, *n* (%)	6 (2.8)	3 (1.4)	0.51	0.098
Reoperation for bleeding, *n* (%)	15 (6.9)	9 (4.2)	0.29	0.118
Myocardial infarction, *n* (%)	2 (0.9)	2 (0.9)	1.00	0.000
Renal failure,^a^ *n* (%)	13 (6.0)	14 (6.5)	1.00	−0.021
Intubation >48 h, *n* (%)	10 (4.6)	6 (2.8)	0.39	0.095
Cardioversion, *n* (%)	9 (4.1)	14 (6.5)	0.38	−0.107
Low cardiac output state, *n* (%)	17 (7.8)	12 (5.5)	0.42	0.092
New-onset cardiac arrythmias				
Atrial fibrillation de novo, *n* (%)	87 (40.1)	76 (35)	0.35	0.105
AV block, *n* (%)	9 (4.1)	8 (3.7)	1.00	0.021
Left bundle branch block, *n* (%)	13 (6.0)	6 (2.8)	0.17	0.157
Right bundle branch block, *n* (%)	6 (2.8)	3 (1.4)	0.45	0.098

Values are represented as *n* (%).

a Renal failure is defined as an elevation >100 μmol/l compared to preoperative level.

AV: atrioventricular; INSPIRIS: INSPIRIS RESILIA; ME: Carpentier Edwards Magna Ease; SMD: standardized mean differences; TIA: transient ischaemic attack.

### Mid-term clinical outcomes

**Figure 1:**
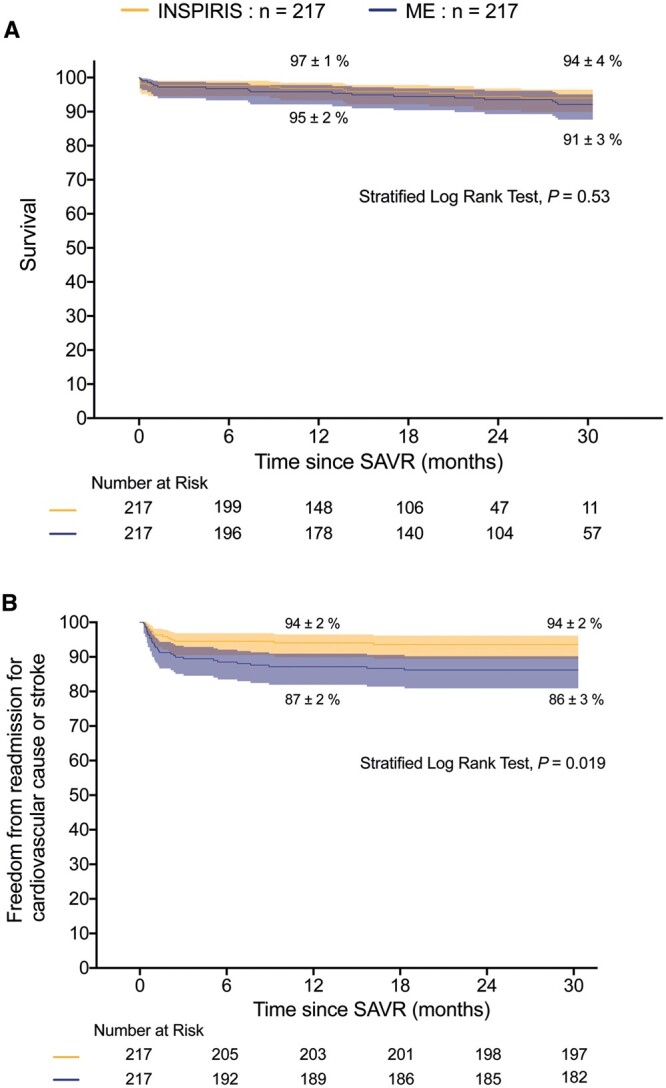
Mid-term outcomes. In the matched cohort, (**A**) survival at 1 year was identical in the both group (INSPIRIS 96.7% vs ME: 95.3%) and at 30 months (INSPIRIS: 93.5% vs ME: 91.2%, *P* overall = 0.53); (**B**) freedom from readmission for cardiovascular causes or stroke/transient ischaemic attack up to 30 months were higher in the INSPIRIS group (93.5% vs 86.2%, *P* = 0.019). INSPIRIS: INSPIRIS RESILIA; ME: Carpentier Edwards Perimount Magna Ease; SAVR: surgical aortic valve replacement.

A total of 33 (8%) deaths and 44 (10%) readmissions occurred in the matched population during a median FU of 19 (12–27) and 18 (9–25) months, respectively. No survival advantage was found at 12 and 30 months in the matched cohort (INSPIRIS: 97% vs ME: 95% and 94% vs 91%, respectively, *P* = 0.53; Fig. 1A). Freedom from readmission for cardiovascular or neurologic cause at 12 and 30 months was however higher in the INSPIRIS group in the matched population (94% vs 87% and 94% vs 86%, respectively, *P* = 0.019; Fig. 1B). Multivariate analyses in the PSM cohort confirmed independent associations between an increase risk in readmission and choice of ME vs. the INSPIRIS (Supplementary Material, Table S2).

There were no aortic valve reinterventions in the INSPIRIS group, compared to 3 reinterventions (0.6%) in the ME group (median time to reintervention of 3.8 [0.9–6.3] months). All reinterventions were for aortic bioprosthesis endocarditis. The FU index for Magna group is 166/217 (77%) and 176/2017 (81%) for INSPIRIS.

### Echocardiographic parameters

**Figure 2:**
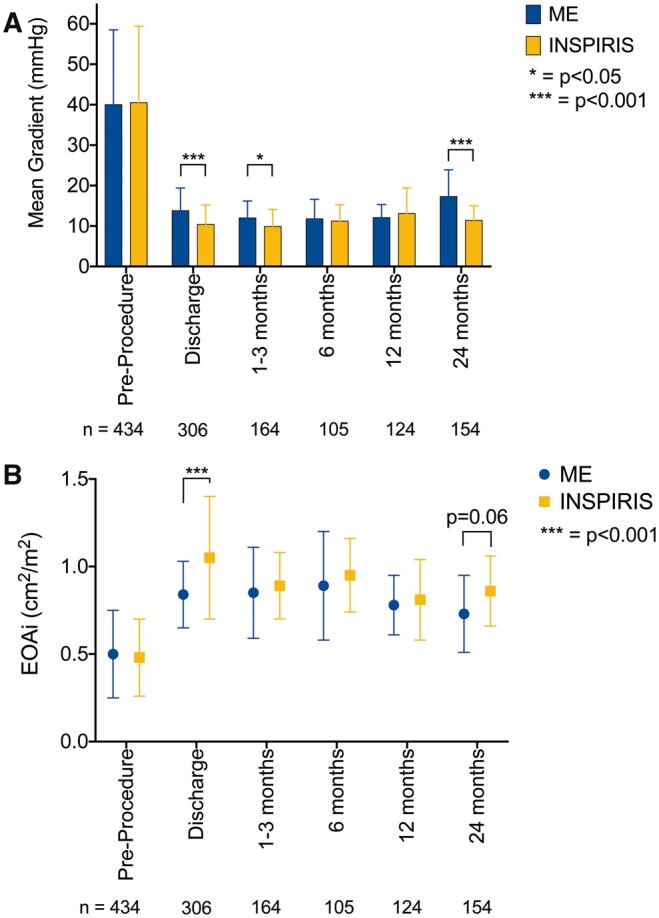
Two-years comparative haemodynamic evaluation of the INSPIRIS and ME valves. In the matched cohort, (**A**) the INSPIRIS valve demonstrated lower gradients at discharge, 1–3 months and 24 months (11.4 [3.6] vs 17.3 [6.6] mmHg, *P* < 0.001, at 24 months) in pairwise analyses. (**B**) In the matched cohort, EOAi followed a similar time course, although it did not reach statistical significance at 1–3 (*P* = 0.60) and 24 months (*P* = 0.06). EOAi: effective orifice area indexed to body surface area. Abbreviations as in Fig. 1.

**Figure 3:**
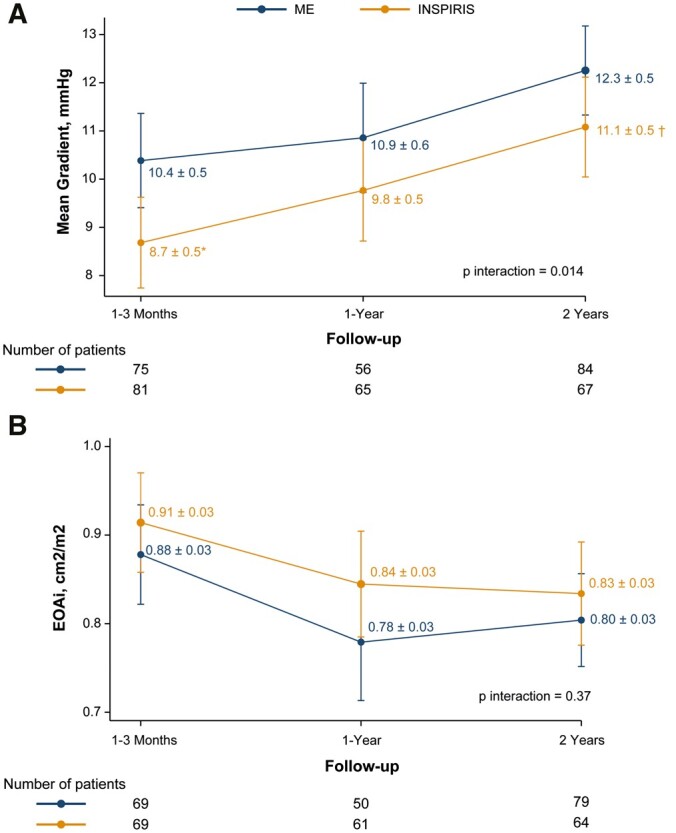
Evolution of haemodynamic performance parameters in follow-up between INSPIRIS and ME valves. In linear mixed-effect models, the INSPIRIS demonstrated significantly better mean gradient (**A**) at 1–3 months and a strong tendency towards significant difference (*P* = 0.06) at 2 years compared to Magna Ease, but no significant differences were observed in EOAi (**B**). Abbreviations as in Figs 1 and 2. *P < 0.05 versus ME valve; ^†^*P* < 0.10 versus ME.

**Figure 4:**
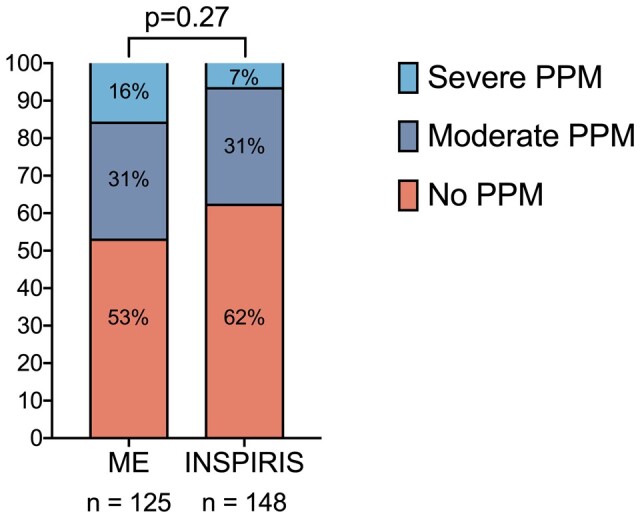
Degree of PPM with the INSPIRIS and ME valves. In the matched cohort, there were numerically more severe PPM in the ME group, although rates were not statistically significant overall (*P* = 0.27). PPM: prosthesis–patient mismatch. Abbreviations as in Figs 1–3.

Both bioprostheses demonstrated good performance and stability over time. In the matched cohort, the INSPIRIS valve demonstrated superior haemodynamics to the ME valve at discharge, 1–3 months and 24 months (Fig. 2A and B, see also Supplementary Material, Table S3). The mean gradients at 24 months in the INSPIRIS group were 11.4 (3.6) mmHg compared to 17.3 (6.6) mmHg in the ME group (*P* < 0.001). Linear mixed-effect analyses are shown in Fig. 3A and B and demonstrate similar results as paired analyses, with a significant difference in MG at 1–3 months and a strong tendency at 24 months between valves. Although there were numerically more severe PPM with the ME valve in the matched population (16% vs 7%), degree of PPM was not statistically different overall between both valves (*P* = 0.27; Fig. 4).

There were 2 events of moderate SVD with the INSPIRIS and none with the ME valve (*P* = 0.87). Both prostheses were 21 mm and SVD was detected at 24 months. These patients did not require reintervention.

## DISCUSSION

The aim of this study was to evaluate the mid-term safety and performance of the INSPIRIS aortic bioprosthesis and benchmark them against those of the ME. The main findings of the current study are as follows: (i) this study represents the largest ‘real-life’ comparative echocardiographic evaluation of the INSPIRIS bioprosthesis; (ii) the INSPIRIS valve demonstrated excellent early postoperative and mid-term clinical outcomes; and (iii) haemodynamics of the INSPIRIS valve are stable through 2-year FU and superior to those of the ME in a propensity score matched cohort.

### Unique characteristics of the INSPIRIS valve and current state of evidence

The RESILIA tissue was first introduced in the Edwards Model 11000A valve (ME with RESILIA tissue). The tissue was initially tested in randomized study on juvenile sheep [13]. Forty-five animals received a ME valve with either standard or treated leaflets in the mitral position. At 8 months, the treated valves demonstrated lower gradients and less calcium content. Model 11000A was then evaluated in 2 large prospective non-randomized single-arm reglementary approval trials. The EU RESILIA feasibility study recruited 133 patients between 2011 and 2013 at 2 sites in Poland [6]. The mean patient age was 65 (13.5) years, including ∼25% of patients under 60. The mean FU was 4.2 years. About 44% of patients received a 19- or 21-mm valve, and no SVD events were reported. The mean gradient and EOA at 5 years was 14.8 (7.6) mmHg and 1.4 (0.5) cm^2^, respectively. The larger COMMENCE trial was a multinational study conducted in 27 centres [5, 7]. From 2013 to 2016, 689 patients were recruited, with a median FU of 4 years. The mean age was 66.9 (11.6) years with 21% of patients under 60 years old. All-cause early and 1- and 4-year mortality were 1.2%, 2.3% and 7.1%, respectively. At 4 years, EOA was 1.5 (0.5) cm^2^ and the mean transvalvular gradient was 11 (5.6) mmHg (∼22% of patients received a 19- or 21-mm valve). There were also no reported cases of SVD.

The INSPIRIS (Model 11500A) combines the RESILIA tissue and the VFit Technology expandable stent frame technological advances. Conceptually, the latter could benefit younger patients, who risk outliving their SAVR, by allowing larger valves/lower gradients in future VinV therapy. However, there is a lack of publicly available data to support this feature. In contrast to Model11000A (ME with RESILIA tissue), there is little evidence in the safety and performance of the INSPIRIS valve. Only a few (4) centres have shared their experience [14–17]. The largest series regroups 100 patients with a 1-year echocardiographic FU [15]. However, most patients were aged <60 years, and 17% received a 19- or 21-mm valve. At 1 year, the mean EOA was 1.8 (0.1) cm^2^ and the mean gradient was 11.5 (2.3) mmHg. The only comparative report that was similar to our study, in that it also compared the INSPIRIS to the ME valve, is the study by Shala *et al.* who presented discharge results in a series of 125 consecutive patients, 59 of whom underwent SAVR with an INSPIRIS valve [16]. In accordance with our findings, postoperative transthoracic echocardiography at 1-week demonstrated lower gradients in favour of the INSPIRIS valve (mean gradient of 10 vs 12 mmHg, *P* < 0.001).

### Clinical outcomes

The current study demonstrated the safety of the INSPIRIS valve at ∼2-year FU. The valve demonstrated excellent postoperative outcomes with a 30-day mortality rate of 1% and 0% stroke rate for isolated AVR procedures. Postoperative outcomes were similar between both valves in the matched population. All-cause mortality at 1 year and 30 months of the ME valve was similar to that of the INSPIRIS valve, which was expected. It should be noted that due to the cost-sharing nature of our healthcare system, and as a general practice, the more expensive INSIPIRIS valve was generally proposed to patients <70 years old. Thus, patients in the INSPIRIS group were generally younger, underwent less combined procedures, and were overall at lower operative risk compared to patients in the ME group. Lower readmission rates with the INSPIRIS valve observed in the matched population may be attributable to the superior haemodynamic performance of the valve, but this hypothesis needs to be validated in further studies. Interaction analyses revealed no significant association with isolated versus non-isolated AVR regarding mid-term outcomes. There were also no reinterventions on the INSPIRIS valve. These results support the mid-term safety of the INSPIRIS valve.

### Haemodynamic performance

Haemodynamics of the INSPIRIS valve were stable throughout FU. In our pairwise analysis, the INSPIRIS demonstrated significantly lower gradients in comparison to the ME valve at discharge (∼10 vs 14 mmHg, *P* < 0.001), 1–3 months (∼10 vs 12 mmHg, *P* < 0.001) and 24-months (∼11 vs 17 mmHg, *P* < 0.001). These results are also comparable to those of other INSPIRIS reports. If this were to hold true in longer-term FU, the INSPIRIS valve may be the better choice. The better haemodynamic performance of the INSPIRIS valve in the first months could be explained by its conceptual design (i.e. smaller sewing cuff and more flexible metallic armature) that generates larger internal diameter than its predecessor (i.e. Magna), but other studies should investigate this hypothesis. This is consistent with Fukunaga *et al.* who evaluate its haemodynamic performance in only 29 Japanese patients treated with the INSPIRIS [17]. Larger studies with larger FU may demonstrate sustained better performance at long term. There were 2 events of moderate SVD in the INSPIRIS group at 2 years and none in the ME group. The difference was not statistically significant and is most likely attributable to the younger patient age in the INSPIRIS group. Interestingly, there were no SVD events reported in the 5-year results of the EU RESILIA and COMMENCE trials [5, 6]. This is most likely attributable to differences in definitions of SVD, long-term FU of the INSPIRIS valve is necessary to better assess this end point. The degree of PPM was similar between both valve types and correlated with what is previously known in the literature [18].

### Future directions

Our single-centre experience with the INSPIRIS valve is growing and longer echocardiographic FU is on-going. We project to share our 5-year clinical outcomes and echocardiographic results in the near future. Within the next ∼5 years, results from large multicentre studies of the INSPIRIS valve/RESILIA tissue with a focus on younger patients should also be available. The Indure Resilia Durability Registry is an open-label, multicentre, European registry with a 5-year FU to assess clinical outcomes in 400 patients under 60 undergoing SAVR with the INSPIRIS [18]. The study was initiated in mid-2019 and is expected to be completed mid-2025. The RESILIENCE trial (NCT03680040) is a US-based study investigating time to valve failure due to valve deterioration requiring reintervention and exploring early potential predictors of valve durability in patients under 65 who underwent SAVR with a RESILIA tissue valve. The study aims to recruit 250 patients which will be followed up to 11 years with an expected completion end in 2027. Interestingly, this study will include valve calcification assessment by multi-slice computerized tomography. The IMPACT registry will recruit 500 patients across 25 EU sites and will have a particular focus on the impact of pre-existing comorbidities on patient outcomes and bioprosthetic valve performance, more specifically, performance of the INSPIRIS valve at a target FU of 5 years [19]. Finally, the INSPIRIS, Valve-in-Valve Surveillance Study (INVIVITY; NCT04902053) should provide initial clues to the potential advantages of the VFit technology.

### Limitations

This study represents the largest echocardiographic comparative analysis of the INSPIRIS and ME bioprosthesis. Some limitations of the current study should be acknowledged. First, this study was retrospective in nature and is thus subject to its inherent limitations. Due to our cost-sharing healthcare system, younger patients were more likely to receive an INSPIRIS valve. The choice between the type of bioprosthesis was not standardized, but operating surgeons had large experience with both valves. The use of PSM has excluded several patients of this initial sample which could limit the generalization of the current findings to the general population. Due to multicentre FU echocardiographic assessment, there may be interobserver variations in assessing EOAi, but the potential variability should be equally distributed in both groups. Further prospective studies should use a standardized assessment of prosthesis measurement and should follow the Valve Labelling Task Force Position Statement [20]. As PPM was evaluated with echocardiographic data, there may be overestimations of the PPM rates, but we encourage the manufacturer to provide standardized charts for the INSPIRIS valve to allow the PPM assessment using the predicted method [20, 21]. In analysing clinical outcomes, the matched population may not have accounted for all potential disparities between the 2 groups due to very different baseline presentation of our patients (e.g. concomitant procedures, as expected), and thus results should be interpreted accordingly. However, multivarible analyses after PSM with the remaining differences confirmed our observations. While clinical FU was complete, there was some missing echocardiographic data that could not be retrieved from referring centres. We however attempted to offer an accurate view of the clinical and echocardiographic comparison of the 2 valves by providing the reader with results from a PSM population of ‘real-life’ patients treated during the same period of time. Unfortunately, our study design was not constructed to evaluate other potential advantages of the INSPIRIS valve, such as the suitability for VinV TAVR. However, future studies should investigate this end point.

## CONCLUSION

This study demonstrated the mid-term safety and haemodynamic performance stability of the INSPIRIS bioprosthesis. The INSPIRIS valve demonstrated lower gradients at 2 years compared to the Magna Ease in a matched population. Rates of SVD and PPM were similar. Long-term clinical and echocardiographic evaluation of the INSPIRIS bioprosthesis compared to the ME is warranted.

## Supplementary Material

ivad117_Supplementary_Data

## Data Availability

The data underlying this article cannot be shared publicly due to government regulations regarding patient data management.

## Notes

Presented at the Rapid Response Session at the 36th EACTS Annual Meeting, Milan, Italy, 5-8 October 2022.

## ABBREVIATIONS

EOA: Effective orifice area
EOAi: Effective orifice area indexed to body surface area
FU: Follow-up
IQR: Interquartile range
INSPIRIS: INSPIRIS RESILIA
ME: Carpentier Edwards Perimount Magna Ease
MG: Mean transvalvular gradient
PPM: Prosthesis–patient mismatch
SAVR: Surgical aortic valve replacement
SVD: Structural valve deterioration
TAVR: Transcatheter aortic valve replacement
VinV: Valve-in-valve
