# Supplementary material for: Mid-term clinical and echocardiographic results of the INSPIRIS RESILIA aortic valve: a retrospective comparison to the Magna Ease
Source: Interdiscip Cardiovasc Thorac Surg. 2023 Jul 18;37(1):ivad117. doi: 10.1093/icvts/ivad117 (PMC10386877; doi:10.1093/icvts/ivad117)
Supplement: ivad117_Supplementary_Data [file ivad117_supplementary_data.docx]

**Supplemental Material for the following article**

**TITLE:** Mid-Term Clinical and Echocardiographic Results of the INSPIRIS Resilia Aortic Valve – A Retrospective Comparison to the Magna Ease

Jérémy Bernard*, MSc; Gabriel Georges*, MD; Sebastien Hecht, MSc; Philippe Pibarot, DVM PhD; Marie-Annick Clavel, DVM PhD; Shervin Babaki, BSc; Dimitri Kalavrouziotis, MD; Siamak Mohammadi, MD

*JB and GG have contributed equally to this work and are thus co-first authors.

**Table of Contents:**

Supplemental Table 1: Page 2

Supplemental Table 2: Page 3

Supplemental Table 3: Page 4

Supplemental Figure 1: Page 5

**Supplemental Table 1.** Post-Operative Outcomes at 30 days Stratified According to Isolated Aortic Valve Replacement

| **Variables** | **Matched Cohort** | | | |
| --- | --- | --- | --- | --- |
|  | **ME**  **(n = 217)** | **INSPIRIS**  **(n = 217)** | **p Value** | **SMD** |
| **Adverse events** |  |  |  |  |
| Isolated AVR | (n = 61) | (n = 84) |  |  |
| In-hospital mortality, n(%) | 1 (1.6) | 0 (0) | 0.63 | 0.180 |
| Stroke/TIA, n(%) | 0 (0) | 0 (0) | 1.00 | 0.000 |
| Cardiogenic shock, n(%) | 2 (3.3) | 0 (0) | 0.18 | 0.261 |
| Reoperation for bleeding, n(%) | 2 (3.3) | 2 (2.4) | 1.00 | 0.054 |
| Myocardial infarction, n(%) | 1 (1.6) | 0 (0) | 0.42 | 0.180 |
| Renal failure*, n(%) | 2 (3.3) | 3 (3.6) | 0.73 | -0.016 |
| Intubation >48h, n(%) | 1 (1.6) | 1 (1.2) | 1.00 | 0.034 |
| Cardioversion, n(%) | 1 (1.6) | 1 (1.6) | 0.75 | 0.000 |
| Low cardiac output state, n(%) | 2 (3.3) | 3 (3.6) | 0.73 | -0.016 |
| Atrial fibrillation de novo, n(%) | 27 (44.3) | 25 (29.8) | 0.26 | 0.304 |
| AV block, n(%) | 2 (3.3) | 3 (3.6) | 0.73 | -0.016 |
| Left bundle branch block, n(%) | 1 (1.6) | 1 (1.2) | 1.00 | 0.034 |
| Right bundle branch block, n(%) | 0 (0) | 0 (0) | 1.00 | 0.000 |
|  |  |  |  |  |
| Non-isolated AVR | (n = 156) | (n = 133) |  |  |
| In-hospital mortality, n(%) | 4 (2.6) | 6 (4.5) | 0.45 | -0.103 |
| Stroke/TIA, n(%) | 5 (3.2) | 4 (3.0) | 1.00 | 0.012 |
| Cardiogenic shock, n(%) | 4 (2.6) | 3 (2.3) | 1.00 | 0.019 |
| Reoperation for bleeding, n(%) | 13 (8.3) | 7 (5.3) | 0.18 | 0.119 |
| Myocardial infarction, n(%) | 1 (0.6) | 2 (1.5) | 0.60 | -0.088 |
| Renal failure*, n(%) | 11 (7.1) | 11 (8.3) | 0.83 | -0.045 |
| Intubation >48h, n(%) | 8 (5.8) | 5 (3.8) | 0.34 | 0.094 |
| Cardioversion, n(%) | 8 (5.1) | 13 (9.8) | 0.55 | -0.180 |
| Low cardiac output state, n(%) | 15 (9.6) | 9 (6.8) | 0.63 | 0.102 |
| Atrial fibrillation de novo, n(%) | 60 (38.5) | 51 (38.3) | 1.00 | 0.004 |
| AV block, n(%) | 7 (4.5) | 5 (3.8) | 0.69 | 0.035 |
| Left bundle branch block, n(%) | 12 (7.7) | 5 (3.8) | 0.11 | 0.168 |
| Right bundle branch block, n(%) | 6 (3.8) | 3 (2.3) | 0.51 | 0.033 |
| Values are n(%). Text in bold highlights the statistically significant differences between the two groups. AV = atrio-ventricular; ME = Carpentier Edwards Magna Ease; INSPIRIS = INSPIRIS RESILIA; TIA = transient ischemic attack. *Renal failure is defined as an elevation >100 umol/L compared to preoperative level. | | | | |

**Supplemental Table 2.** Cox Analysis of All-cause Mortality and Readmission in Propensity-Score Matched Cohort

| **Variables** | **All-cause Mortality (33 events)** | | | | **Cardiovascular Readmission (44 events)** | | | |
| --- | --- | --- | --- | --- | --- | --- | --- | --- |
|  | **Univariable analysis** | | **Multivariate analysis*** | | **Univariable analysis** | | **Multivariate analysis*** | |
|  | **HR (95% CI)** | **p Value** | **HR (95% CI)** | **p Value** | **HR (95% CI)** | **p Value** | **HR (95% CI)** | **p Value** |
| ME vs. INSPIRIS | 1.67 (0.70 – 3.96) | 0.25 | 0.78 (0.25 – 2.40) | 0.66 | 2.18 (1.15 – 4.10) | **0.02** | 2.81 (1.26 – 6.26) | **0.01** |
| **Bold** indicates statistical significance. CI = confidence interval; HR = hazard ratio; ME = Carpentier Edwards Magna Ease; INSPIRIS = INSPIRIS RESILIA  * Adjusted for the main remaining differences after propensity-score in Tables 1 et 2 of the manuscript (i.e., isolated aortic valve replacement [concomitant procedures], aortic valve etiology, left ventricular stroke volume and systolic pulmonary artery pressure). | | | | | | | | |

**Supplemental Table 3.** Valve Hemodynamics Over Time in Propensity Score Matched Cohort

|  | **Pre-Procedure**  **(n = 434)** | | | **Discharge**  **(n = 306)** | | | **1-3 months**  **(n = 164)** | | |
| --- | --- | --- | --- | --- | --- | --- | --- | --- | --- |
|  | **ME**  **(n = 217; 50%)** | **INSPIRIS**  **(n = 217; 50%)** | **P Value** | **ME**  **(n = 213; 50%)** | **INSPIRIS**  **(n = 213; 50%)** | **P Value** | **ME**  **(n = 79; 48%)** | **INSPIRIS**  **(n = 85; 52%)** | **P Value** |
| Mean gradient, mmHg | 40.0 (18.5)  (n = 206) | 40.5 (18.9)  (n = 206) | 0.81 | 13.8 (5.6)  (n = 153) | 10.4 (4.8)  (n = 153) | **<0.001** | 12.0 (4.2)  (n = 34) | 9.9 (4.2)  (n = 34) | **0.02** |
| EOAi, cm^2^/m^2^ | 0.50 (0.25)  (n = 192) | 0.48 (0.22)  (n = 192) | 0.40 | 0.84 (0.19)  (n = 80) | 1.05 (0.35)  (n = 80) | **<0.001** | 0.85 (0.26)  (n = 24) | 0.89 (0.19)  (n = 24) | 0.60 |
|  | **6 months**  **(n = 105)** | | | **1 year**  **(n = 124)** | | | **2-3 years**  **(n = 154)** | | |
|  | **ME**  **(n = 43; 41%)** | **INSPIRIS**  **(n = 62; 59%)** | **P Value** | **ME**  **(n = 58; 47%)** | **INSPIRIS**  **(n = 66; 53%)** | **P Value** | **ME**  **(n = 85; 55%)** | **INSPIRIS (n = 69; 45%)** | **P Value** |
| Mean gradient, mmHg | 11.8 (4.8)  (n = 13) | 11.2 (4.08)  (n = 13) | 0.64 | 12.1 (3.2)  (n = 18) | 13.1 (6.3)  (n = 18) | 0.54 | 17.3 (6.6)  (n = 25) | 11.4 (3.6)  (n = 25) | **<0.001** |
| EOAi, cm^2^/m^2^ | 0.89 (0.31)  (n = 11) | 0.95 (0.21)  (n = 11) | 0.49 | 0.78 (0.17)  (n = 16) | 0.81 (0.23)  (n = 16) | 0.64 | 0.73 (0.22)  (n = 24) | 0.86 (0.20)  (n = 24) | 0.063 |
| Values are mean (SD). EOAi = effective orifice area indexed to body surface area; ME = Carpentier Edwards Perimount Magna Ease; INSPIRIS = Inspiris Resilia | | | | | | | | | |

**Supplementary Figure 1.** Study Design


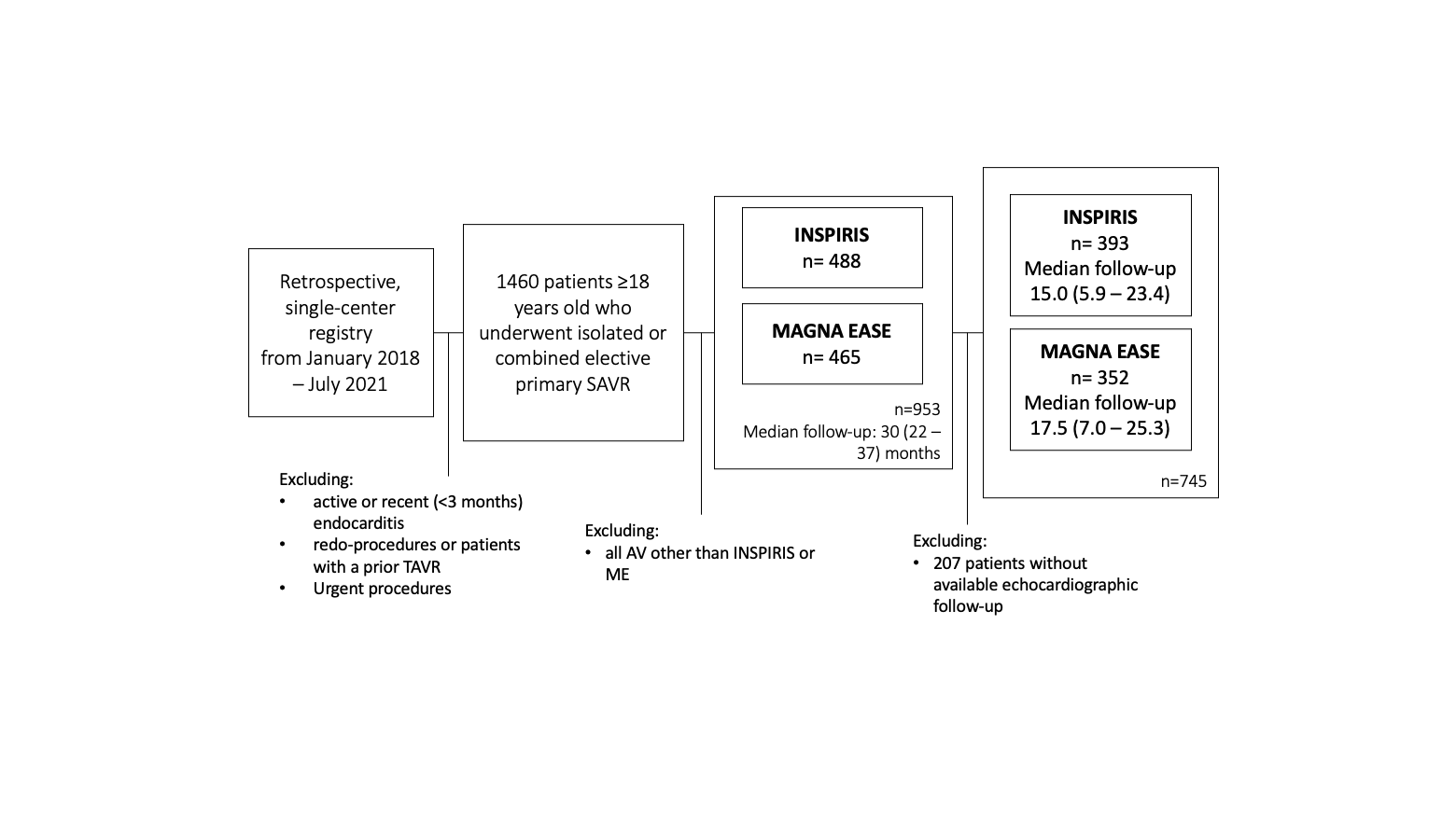


**Legend:** There were 953 patients included in this study, of which 488 received an INSPIRIS bioprosthetic aortic valve. Of all patients, 745 had at least one echocardiographic follow-up, whit a median follow-up of 30 months (IQR: 22-37 months). ME = Carpentier Edwards Perimount Magna Ease, INSPIRIS = INSPIRIS RESILIA
